# Evidences for the Involvement of Monoaminergic and GABAergic Systems in Antidepressant-like Activity of *Tinospora cordifolia* in Mice

**DOI:** 10.4103/0250-474X.49118

**Published:** 2008

**Authors:** D. Dhingra, P. K. Goyal

**Affiliations:** Department of Pharmaceutical Sciences, Guru Jambheshwar University of Science and Technology, Hisar-125001, India

**Keywords:** Depression, forced swim test, monoamine oxidase, tail suspension test, *Tinospora cordifolia*

## Abstract

The present study was taken up to investigate the effect of petroleum ether extract of *Tinospora cordifolia* (Wild.) Miers, on depression in mice. The extract (50, 100 and 200 mg/kg, p.o.) was administered for 14 successive days to Swiss young albino mice (either sex) and evaluated for antidepressant-like activity using tail suspension test and forced swim test. Petroleum ether extract at all three doses produced significant antidepressant-like effect in tail suspension test as well as in forced swim test and their efficacies were found to be comparable to imipramine (15 mg/kg, p.o.) and sertraline (20 mg/kg, p.o.). The extract at a dose of 50 mg/kg showed most potent effect and did not show any significant change in locomotor functions of mice as compared to control. The antidepressant-like effect of the extract was significantly reversed by pretreatment of animals with prazosin (a α_1_-adrenoceptor antagonist), sulpiride (a selective dopamine D_2_-receptor antagonist), p-CPA (a serotonin synthesis inhibitor) and baclofen (GABA-B agonist), when tested in tail suspension test. Moreover, petroleum ether extract also reduced the mouse whole brain monoamine oxidase (MAO-A and MAO-B) activities as compared to control, resulting in increase in the levels of brain monoamines. Therefore, the extract may have potential therapeutic value for the management of depressive disorders.

Mental depression represents a major public health problem worldwide. The high prevalence of suicide in depressed patients (up to 15%) coupled with complications arising from stress and its effects on the cardiovascular system have suggested that it will be the second leading cause of death by the year 2020^1^. The use of alternative medicines is increasing worldwide day by day. *Hypericum perforatum*, a well known plant has been proven to be effective antidepressant in clinical studies^2^. Thus, there is a constant need to identify newer natural antidepressants with greater efficacy, and to explore their potential over synthetic antidepressants. *Tinospora cordifolia* (Family: Menispermaceae), a well known plant of Indian medicinal system, was selected for evaluating antidepressant-like activity in laboratory animals, since this plant has been reported to possess antistress activity^3^,^4^. According to the Ayurvedic System of Medicine, *T. cordifolia* is an antigout, analgesic, rejuvenator, astringent, anthelmintic, antiarthritic, antiperiodic, antipyretic, antimalarial, antiinflammatory, aphrodisiac, antiasthmatic, bitter tonic, carminative, cardiotonic, constipative, digestant, diuretic, blood purifier, expectorant, antidiabetic, antigonorrhoeal, cholagogue, antiemetic and antiicteric^5^–^7^. *T. cordifolia* has been claimed to possess learning and memory enhancing^8^, antioxidant^9^, antiischemic^10^, hypolipidaemic^11^, antidiabetic^12^, antiulcer^13^, hepatoprotective^14^, antifertility^15^, antiinflammatory^16^, antiallergic^17^, immunomodulatory^18^, anticancer^19^ and radioprotective^20^. The chemical constituents of *T. cordifolia* stems include alkaloids like berberine, palmatine, tembetarine, magnoflorine^21^,^22^, glycosides like tinocordiside^23^, tinocordifolioside, cordioside^24^, cordifolioside A, B^25^, cordifoliside A, B, C, D, E^26^,^27^, steroids like ecdysterone, makisterone A, giloinsterol^28^, sesquiterpenoids like tinocordifolin^29^. Thus, the aim of the present study was to explore the antidepressant potential of *T. cordifolia* and to investigate the probable underlying mechanisms of action.

## MATERIALS AND METHODS

Swiss mice of either sex, weighing around 20-25 g were purchased from Disease-free Small Animal House, Chaudhary Charan Singh Haryana Agricultural University, Hisar (Haryana, India). Male and female animals were housed separately in groups of 6 per cage (polycarbonate cage size: 29×22×14 cm) under laboratory conditions with alternating light and dark cycle of 12 h each. The animals had free access to food and water. The animals were kept fasted 2 h before and 2 h after drug administration. The animals were acclimatized for at least five days before behavioral experiments which were carried out between 09:00 and 17:00 h. The experimental protocol was approved by Institutional Animals Ethics Committee (IAEC) and animal care was taken as per the guidelines of Committee for the Purpose of Control and Supervision of Experiments on Animals (CPCSEA), Govt. of India (Registration No. 0436).

Sertraline hydrochloride (Aurobindo Pharma Ltd., Hyderabad, India), prazosin hydrochloride, (±) sulpiride, DL para-chlorophenylalanine, baclofen and imipramine hydrochloride (Sigma-Aldrich, St. Louis, USA); Tween 80 (Loba Chemie, Mumbai, India); petroleum ether (60-80°), Tris (hydroxymethyl)aminomethane, benzylamine (S. D. Fine-Chem Ltd., Mumbai, India), di-sodium hydrogen phosphate (Merck Ltd, Mumbai, India), sodium di-hydrogen orthophosphate, sucrose, acetic acid, hydrochloric acid (Qualigens Fine Chemicals, Mumbai, India), EDTA di-sodium salt, sodium hydroxide pellets, 5-hydroxy tryptamine, creatine sulphate (Hi-Media Laboratories Pvt. Ltd., Mumbai, India), bovine serum albumin (Spectrochem Pvt. Ltd., Mumbai, India) were used in the present study.

### Collection of plant material:

The dried stems of *T. cordifolia* were purchased from the commercial market, New Delhi and were authenticated as *Tinospora cordifolia* (Wild.) Miers ex Hook. f. and Thoms from Raw Materials Herbarium and Museum section of National Institute of Science Communication and Information Resources, New Delhi (Ref. No. NISCAIR/RHMD/Consult/06/741/58).

### Preparation of petroleum ether extract:

The dried stems were grounded to coarse powder. About 1kg of powdered drug was extracted with petroleum ether (60-80°) using Soxhlet apparatus at 70° till siphoning solution became colorless. The solvent was recovered by distillation and the extract was dried by using water bath at 50-60°. The dried petroleum ether extract was yellowish in color and the yield was 0.89%. The dried extract was stored in air tight container and kept in a refrigerator.

### Preliminary phytochemical screening:

For preliminary phytochemical screening, petroleum ether extract was tested for the presence of alkaloids, glycosides, carbohydrates, sterols, phenolic compounds and tannins, flavonoids, saponins, proteins and amino acids following the standard procedures^30^. The extract of *T. cordifolia* was emulsified in 10% v/v Tween 80. Imipramine, sertraline, prazosin and baclofen were separately dissolved in normal saline (0.9% NaCl). Sulpiride was dissolved in normal saline followed by the addition of one drop of glacial acetic acid. p-CPA was dissolved in minimum quantity of 0.1N sodium hydroxide solution and pH was adjusted to 7.0 with 0.1N hydrochloric acid^31^.

### Tail suspension test (TST):

TST, commonly employed behavioral model for screening antidepressant-like activity in mice, was first discovered by Steru *et al.*^32^. The test was conducted as previously followed^31^. Animals were moved from their housing colony to laboratory in their own cages and allowed to adapt to the laboratory conditions for 1-2 h. Each mouse was individually suspended to the edge of a table, 50 cm above the floor, by adhesive tape placed approximately 1 cm from the tip of the tail. Each animal under test was both acoustically and visually isolated from other animals during test. The total period of immobility was recorded manually for 6 min. Animal was considered to be immobile when it didn't show any body movement, hung passively and completely motionless. The test was conducted in a dim lighted room and each mouse was used only once in the test. The observer, recording the immobility of animals, was blind to the drug treatments given to the animals under study.

### Forced swimming test (FST):

FST, the most frequently used behavioral model for screening antidepressant-like activity in rodents, was first proposed by Porsolt *et al*^33^. The procedure was same as previously followed^31^. Animals were moved from their housing colony to laboratory in their own cages and allowed to adapt to the laboratory conditions for 1-2 h. Mice were individually forced to swim in open glass chamber (25×15×25 cm^3^) containing fresh water to a height of 15 cm and maintained at 26±1°. Water in the chamber was changed after subjecting each animal to FST because used-water has been shown to alter the behavior^34^. Each animal showed vigorous movement during initial 2 min period of the test. The duration of immobility was manually recorded during the next 4 min of the total 6 min testing period. Mice were considered to be immobile when they ceased struggling and remained floating motionless in water, making only those movements necessary to keep their head above water. The test was conducted in a dim lighted room and each mouse was used only once in the test^35^. The observer, recording the immobility of animals, was blind to the drug treatments given to the animals under study.

### Measurement of MAO-A and MAO-B:

On 14^th^ day, mice were sacrificed after 6 min exposure to FST, and the brain samples were collected immediately on a ice plate. The collected brain samples were washed with cold 0.25M Sucrose, 0.1M Tris, 0.02M EDTA buffer (pH 7.4) and weighed. The whole procedure of brain isolation was completed within five minutes^36^–^37^. Mouse brain mitochondrial fractions were prepared following the procedure of Schurr and Livne^36^. The MAO activity was assessed spectrophotometrically^37^–^39^. Briefly, the buffer washed brain sample was homogenized in 9 volumes of cold 0.25 M sucrose, 0.1 M Tris, 0.02 M EDTA buffer (pH 7.4) buffer and centrifuged twice at 800 g for 10 min at 4° in cooling centrifuge (Remi instruments, Mumbai). The pellet was discarded. The supernatant was then centrifuged at 12000 g for 20 min at 4° in cooling centrifuge. The precipitates were washed twice with about 100 ml of sucrose-Tris-EDTA buffer and suspended in 9 volumes of cold sodium phosphate buffer (10 mM, pH 7.4, containing 320 mM sucrose) and mingled well at 4° for 20 min. The mixture was then centrifuged at 15000 g for 30 min at 0° and the pellets were re-suspended in cold sodium phosphate buffer. The protein concentration was estimated by Lowry method using bovine serum albumin as the standard^40^. The assay mixture contained 100 μl of 4 mM 5-hydroxytryatpamine and 100 μl of 0.1 M benzylamine as the specific substrate for MAO-A and MAO-B, respectively, 150 μl solution of mitochondrial fraction and 2.75 ml sodium phosphate buffer (100 mM, pH 7.4).

For estimating MAO-B activity, 2.75 ml sodium phosphate buffer (100 mM, pH 7.4) and 100 μl of 0.1 M benzylamine were mixed in a quartz cuvette which was then placed in double beam spectrophotometer (Systronics 2203, Bangalore, India). This was followed by the addition of 150 μl solution of mitochondrial fraction to initiate the enzymatic reaction and the change in absorbance was recorded at wavelength of 249.5 nm for 5 min against the blank containing sodium phosphate buffer and benzylamine.

For estimating MAO-A activity, 2.75 ml sodium phosphate buffer (100 mM, pH 7.4) and 100 μl of 4 mM 5-hydroxytryptamine were mixed in a quartz cuvette which was then placed in double beam spectrophotometer (Systronics 2203, Bangalore, India). This was followed by the addition of 150 μl solution of mitochondrial fraction to initiate the enzymatic reaction and the change in absorbance was recorded at wavelength of 280 nm for 5 min against the blank containing sodium phosphate buffer and 5-hydroxytryptamine.

### Measurement of locomotor activity:

To rule out the effects of the extract on immobility period, horizontal locomotor activities of control and test animals were recorded for a period of 10 min using Medicraft Photoactometer- Model No. 600-4D (INCO, Ambala, India).

Animals were divided into 25 groups and each group comprised of a minimum of 6 mice. The experimental protocol was divided into the following parts:

### Antidepressant-like activity using tail suspension test:

In group 1 (control group), 10% v/v Tween 80 was administered orally for 14 consecutive days and on 14^th^ day, 60 min after the administration; immobility period was recorded in TST. In groups 2 and 3, imipramine (15 mg/kg) and sertraline (20 mg/kg) respectively were orally administered for 14 successive days and on 14^th^ day, 60 min after the administration; immobility period was recorded in TST. In groups 4, 5 and 6, petroleum ether extract (50, 100 and 200 mg/kg, p.o. respectively) of *T. cordifolia* was administered for 14 successive days and on 14^th^ day, 60 min after the administration, immobility period was recorded in TST.

### Antidepressant-like activity using forced swim test:

Group 7 to 12 were similar as mentioned under TST (Group 1 to 6) except that the immobility period was recorded using FST. Thus, group 7 was similar to group 1; group 8 was similar to group 2 and so on.

### Mechanisms of action studies in tail suspension test:

In group 13 (sulpiride control), vehicle (10% v/v Tween 80) was administered orally for 14 consecutive days and on 14^th^ day, after 45 min of vehicle treatment; sulpiride (50 mg/kg, i.p.) was injected. After 45 min of injection, the animals were subjected to tail suspension test. In group 14, petroleum ether extract (50 mg/kg, p.o.) of *T. cordifolia* was administered for 14 consecutive days and on 14^th^ day, after 45 min of extract treatment; sulpiride (50 mg/kg, i.p.) was injected and the animals were subjected to tail suspension test after 45 min of the injection. In group 15 (baclofen control), vehicle (10% v/v Tween 80) was administered orally for 14 consecutive days and on 14^th^ day, after 45 min of vehicle treatment; baclofen (10 mg/kg, i.p.) was injected. After 45 min of injection, the animals were subjected to tail suspension test. In group 16, petroleum ether extract (50 mg/kg, p.o.) of *T. cordifolia* was administered for 14 consecutive days and on 14^th^ day, after 45 min of extract treatment; baclofen (10 mg/kg, i.p.) was injected and the animals were subjected to tail suspension test after 45 min of the injection. In group 17 (prazosin control), vehicle (10% v/v Tween 80) was administered orally for 14 consecutive days and on 14^th^ day, after 45 min of vehicle treatment; prazosin (62.5 μg/kg, i.p.) was injected. After 45 min of injection, the animals were subjected to tail suspension test. In group 18, petroleum ether extract (50 mg/kg, p.o.) of *T. cordifolia* was administered for 14 consecutive days and on 14^th^ day, after 45 min of extract treatment; prazosin (62.5 μg/kg, i.p.) was injected and the animals were subjected to tail suspension test after 45 min of the injection. In group 19 (p-CPA control), vehicle (10% v/v Tween 80) was administered orally for 14 consecutive days. pCPA (100 mg/kg, i.p.) was injected from 11^th^ to 14^th^ day after 45 min of vehicle treatment. On 14^th^ day, the animals were subjected to tail suspension test after 45 min of pCPA injection. In group 20, petroleum ether extract (50 mg/kg, p.o.) of *T. cordifolia* was administered for 14 consecutive days. pCPA (100 mg/kg, i.p.) was injected from 11^th^ to 14^th^ day after 45 min of vehicle treatment. On 14^th^ day, the animals were subjected to tail suspension test after 45 min of pCPA injection.

### Estimation of brain MAO:

In group 21, vehicle (10% v/v Tween 80) was administered orally for 14 successive days and on 14^th^ day, 60 min after the administration; the animals were subjected to forced swim test for 6 min, and immediately brain samples were collected. MAO-A and MAO-B levels were measured in the brain samples. In group 22, imipramine (15 mg/kg, p.o.) was administered orally for 14 successive days and on 14^th^ day, 60 min after the administration; the animals were subjected to forced swim test for 6 min, and immediately brain samples were collected. MAO-A and MAO-B levels were measured in the brain samples. In group 23, petroleum ether extract (50 mg/kg, p.o.) was administered orally for 14 successive days and on 14^th^ day, 60 min after the administration; the animals were subjected to forced swim test for 6 min, and immediately brain samples were collected. MAO-A and MAO-B levels were measured in the brain samples.

### Measurement of locomotor activity:

In group 24, vehicle (10% v/v Tween 80) was administered orally for 14 successive days and on 14^th^ day, 60 min after the administration; locomotor activity was measured. In group 25, petroleum ether extract (50 mg/kg, p.o.) of *T. cordifolia* were administered for 14 consecutive days and on 14^th^ day, 60 min after the administration; locomotor activity was measured.

### Statistical analysis:

All the results were expressed as mean±standard error mean (SEM). The data of all the groups were analyzed using one-way ANOVA followed by Dunnett's t-test using the software Sigma-Stat 3.5. The data for locomotor activity scores was subjected to Student's unpaired t-test. In all the tests, the criterion for statistical significance was *P*<0.05.

## RESULTS

The results of phytochemical screening indicated the presence of alkaloids, glycosides, carbohydrates, sterols and flavonoids in petroleum ether extract.

### Effect of petroleum ether extract on immobility periods in TST and FST:

Petroleum ether extract (50, 100 and 200 mg/kg, p.o.) administered for 14 successive days to mice significantly decreased the immobility periods in both TST and FST, indicating significant antidepressant-like activity. Among three doses, the dose of 50 mg/kg of petroleum ether extract decreased the immobility period to the greatest extent, thus showed most potent antidepressant-like action. Imipramine (15 mg/kg, p.o.) and sertraline (20 mg/kg, p.o.) administered for 14 successive days to mice significantly decreased the immobility periods in both TST and FST as compared to control, thus showed significant antidepressant-like action (Tables 1 and 2).

**TABLE 1:** EFFECT OF *TINOSPORA CORDIFOLIA* ON IMMOBILITY PERIOD OF MICE USING TAIL SUSPENSION TEST

Group No.	Treatment for 14 days p.o.	Dose (kg-1)	Immobility Period (s)
1	Vehicle	10 ml	166.5±7.9
2	Imipramine	15 mg	112.8±3.9*
3	Sertraline	20 mg	79.8±6.4*
4	Petroleum ether extract	50 mg	107.3±6.3*
5	Petroleum ether extract	100 mg	112.2±6.6*
6	Petroleum ether extract	200 mg	121.3±4.6*

n=6 in each group; Values are in mean±SEM. Data was analyzed by one-way ANOVA followed by Dunnett's t-test. F(5,30)= 21.45; *P*< 0.05

* *P*< 0.05 when compared with vehicle treated group.

**TABLE 2:** EFFECT OF *TINOSPORA CORDIFOLIA* ON IMMOBILITY PERIOD OF MICE USING FORCED SWIM TEST

Group No.	Treatment for 14 days p.o.	Dose (kg-1)	Immobility Period (s)
7	Vehicle	10 ml	158.2±5.1
8	Imipramine	15 mg	109.8±7.7*
9	Sertraline	20 mg	106.6±4.9*
10	Petroleum ether extract	50 mg	82.3±5.5*
11	Petroleum ether extract	100 mg	95.5±8.4*
12	Petroleum ether extract	200 mg	97.0±7.6*

n=6 in each group; Values are in mean±SEM. Data was analyzed by one-way ANOVA followed by Dunnett's t-test. F(5,30)= 15.51; *P*< 0.05

* *P*< 0.05 when compared with vehicle treated group.

### Effect of combination of petroleum ether extract with sulpiride, baclofen, prazosin and *P*-CPA on immobility period in TST:

Sulpiride (50 mg/kg, i.p.), baclofen (10 mg/kg, i.p.) prazosin (62.5 μg/kg, i.p.) and *p*-CPA (100 mg/kg, i.p.) alone significantly increased the immobility period as compared to control group. Pretreatment of animals with sulpiride or baclofen or prazosin or *p*-CPA significantly reversed the decrease in immobility time elicited by petroleum ether extract at the dose 50 mg/kg (Table 3).

**TABLE 3:** EFFECTS OF SULPIRIDE, BACLOFEN, PRAZOSIN AND *p*-CPA ON ANTIDEPRSSANT-LIKE ACTIVITY OF *TINOSPORA CORDIFOLIA* IN TAIL SUSPENSION TEST

Group No.	Treatment for 14 days p.o.	Dose (kg-1)	Immobility Period (s)
1	Vehicle	10 ml	166.5±7.9
4	Petroleum ether extract	50 mg	107.3±6.3
13	Vehicle + Sulpiride	10 ml+50 mg	201.2±9.2*
14	Petroleum ether extract + Sulpiride	50 mg+50 mg	198.3±11.9#
15	Vehicle + Baclofen	10 ml+10 mg	231.8±7.2*
16	Petroleum ether extract + Baclofen	50 mg+10 mg	136.2±9.9#
17	Vehicle + Prazosin	10 ml+62.5 μg	203.2±5.9*
18	Petroleum ether extract + Prazosin	50 mg+62.5 μg	183.0±6.1#
19	Vehicle + p-CPA	10 ml+100 mg	212.8±4.9*
20	Petroleum ether extract + p-CPA	50 mg+100 mg	133.0±5.3#

n=6 in each group; Values are in mean±SEM. Data was analyzed by one-way ANOVA followed by Dunnett's t-test. F(4,25)= 10.989; *P*< 0.05 (for vehicle treated groups 1, 13, 15, 17 and 19).

* *P*< 0.05 when compared with vehicle treated group (1). F(4,25)= 20.607; *P*< 0.05 (for  petroleum ether extract treated groups 4, 14, 16, 18 and 20).

# *P*< 0.05 when compared with petroleum ether extract treated group (4).

### Effect of petroleum ether extract on brain monoamine oxidase (MAO) activities:

**Fig. 1:**
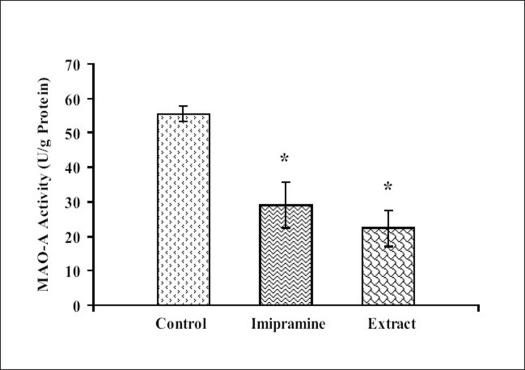
Effect of petroleum ether extract of *T. cordifolia* on MAO-A activity n=6 in each group; Values are in mean±SEM. Data was analyzed by one-way ANOVA followed by Dunnett's t-test. F (2,15)= 12.235; *P*<0.05, *P<0.05 when compared with vehicle treated group.

**Fig. 2:**
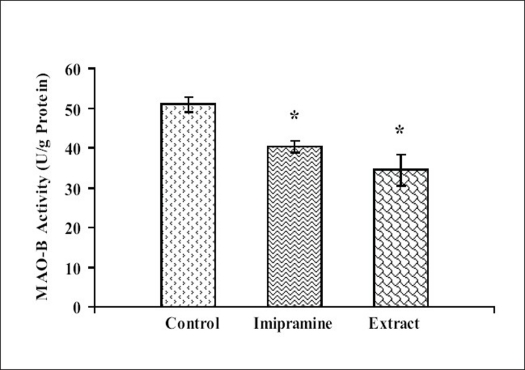
Effect of petroleum ether extract of *T. cordifolia* on MAO-B activity n=6 in each group; Values are in mean±SEM. Data was analyzed by one-way ANOVA followed by Dunnett's t-test. F(2,15)= 9.545; *P*<0.05, *P<0.05 when compared with vehicle treated group.

Petroleum ether extract (50 mg/kg) administered for 14 consecutive days to mice, significantly reduced the brain MAO-A and MAO-B levels as compared to the respective vehicle treated groups. The efficacy of petroleum ether extract, was found to be comparable to imipramine (Figs. 1 and 2).

### Effect on locomotor activity:

Petroleum ether extract (50 mg/kg, p.o.) administered for 14 successive days did not show any significant change in the locomotor function of mice (642.8 ± 84.5) as compared to the vehicle treated group (737.3 ± 50.1).

## DISCUSSION

In the present study, petroleum ether extract (50, 100 and 200 mg/kg, p.o.) administered for 14 successive days to mice produced significant antidepressant-like effect in TST as well as in FST. The efficacy of the extract was found to be comparable to imipramine (15 mg/kg, p.o.) and sertraline (20 mg/kg, p.o.). FST and TST are two commonly used behavioral despair models of depression. These models are widely employed in rodents to predict antidepressant potential by decrease of immobility period produced by several different classes of antidepressant drugs^32^–^33^. Petroleum ether extract at the dose of 50 mg/kg, p.o. (most effective dose) did not show any significant change in locomotor functions of mice as compared to control, so it did not produce any motor effects. It confirmed the assumption that the antidepressant-like effect of the extract was specific and not the false positive. The precise mechanisms by which petroleum ether extract of *T. cordifolia* produced antidepressant-like effect are not completely understood. However according to our results, the antidepressant-like effect of the extract was significantly reversed by pretreatment of animals with prazosin (a α_1_-adrenoceptor antagonist), sulpiride (a selective dopamine D_2_-receptor antagonist), *p*-CPA (a serotonin synthesis inhibitor) and baclofen (GABA_B_ agonist), when tested in TST. This suggested that the petroleum ether extract might produce antidepressant-like effect by interaction with α_1_-adrenoceptors, dopamine D_2_-receptors, serotonergic and GABA_B_ receptors, hence increasing the levels of norepinephrine, dopamine and serotonin; and decreasing the levels of GABA in brains of mice. Levels of monoamines like norepinephrine, serotonin and dopamine are decreased in depression, so antidepressant drugs enhance the levels of these monoamines. GABA_B_ receptor antagonism may serve as a basis for the generation of novel antidepressants^41^.

Moreover, petroleum ether extract also reduced the mouse whole brain MAO-A and MAO-B activities as compared to control, so it indicated that this extract inhibited the metabolism of monoamines, particularly serotonin and noradrenaline. Monoamine oxidase (MAO) regulates the metabolic degradation of catecholamines, serotonin and other endogenous amines in CNS. Inhibition of this enzyme causes a reduction in metabolism and subsequent increase in the concentration of biogenic amines. MAO-A preferentially metabolize adrenaline, nor-adrenaline and serotonin. MAO-B metabolizes phenylethylamines. Dopamine is metabolized by both MAO-A and MAO-B^42^.

Thus, petroleum ether extract of *T. cordifolia* showed antidepressant-like activity probably by inhibiting MAO-A and MAO-B, thus increasing the levels of monoamines like noradrenaline, serotonin, and dopamine; and decreasing the levels of GABA. This was also supported by the earlier study where petroleum ether extract of *T. cordifolia* showed anti-stress activity^4^. According to results of phytochemical screening and literature, the antidepressant-like action of petroleum ether extract might be due to the presence of berberine (alkaloid), since the latter compound has been reported to have antidepressant-like activity^43^, however the role of other constituents present in petroleum ether extract towards antidepressant-like activity needs to be explored. Therefore, *T. cordifolia* extract may have potential therapeutic value for the management of depressive disorders.
